# Supplementary material for: Early-diverging plesiosaurs from the Pliensbachian (Lower Jurassic) of northwestern Germany
Source: PeerJ. 2024 Nov 26;12:e18408. doi: 10.7717/peerj.18408 (PMC11606318; doi:10.7717/peerj.18408)

Supplementary Information 3 for:

**Early-diverging plesiosaurs from the Pliensbachian (Lower Jurassic) of northwestern Germany**

Sven Sachs, Jahn J. Hornung & Daniel Madzia

**Figure S1.** Parsimony analysis using equal weights. Strict consensus tree. Numbers on nodes show Bremer support values.


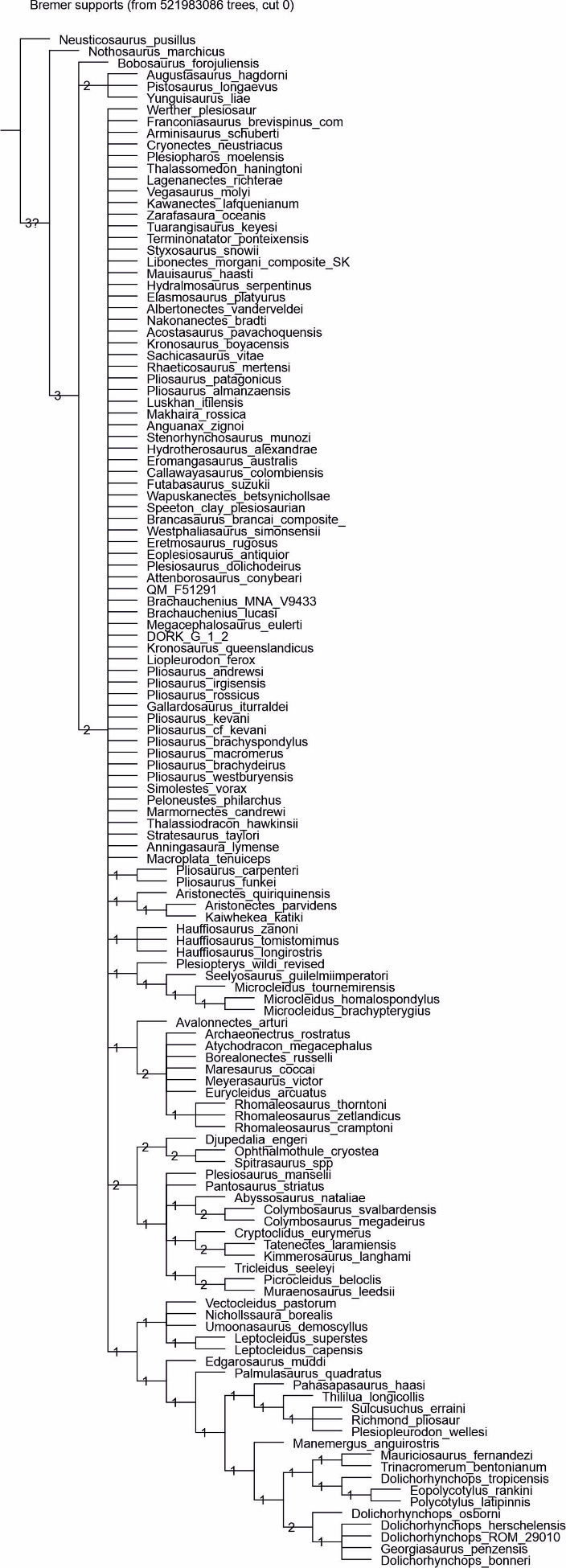


**Figure S2.** Parsimony analysis using equal weights. Majority rule consensus tree. Numbers on nodes show the percentage of the most parsimonious trees that found the nodes.


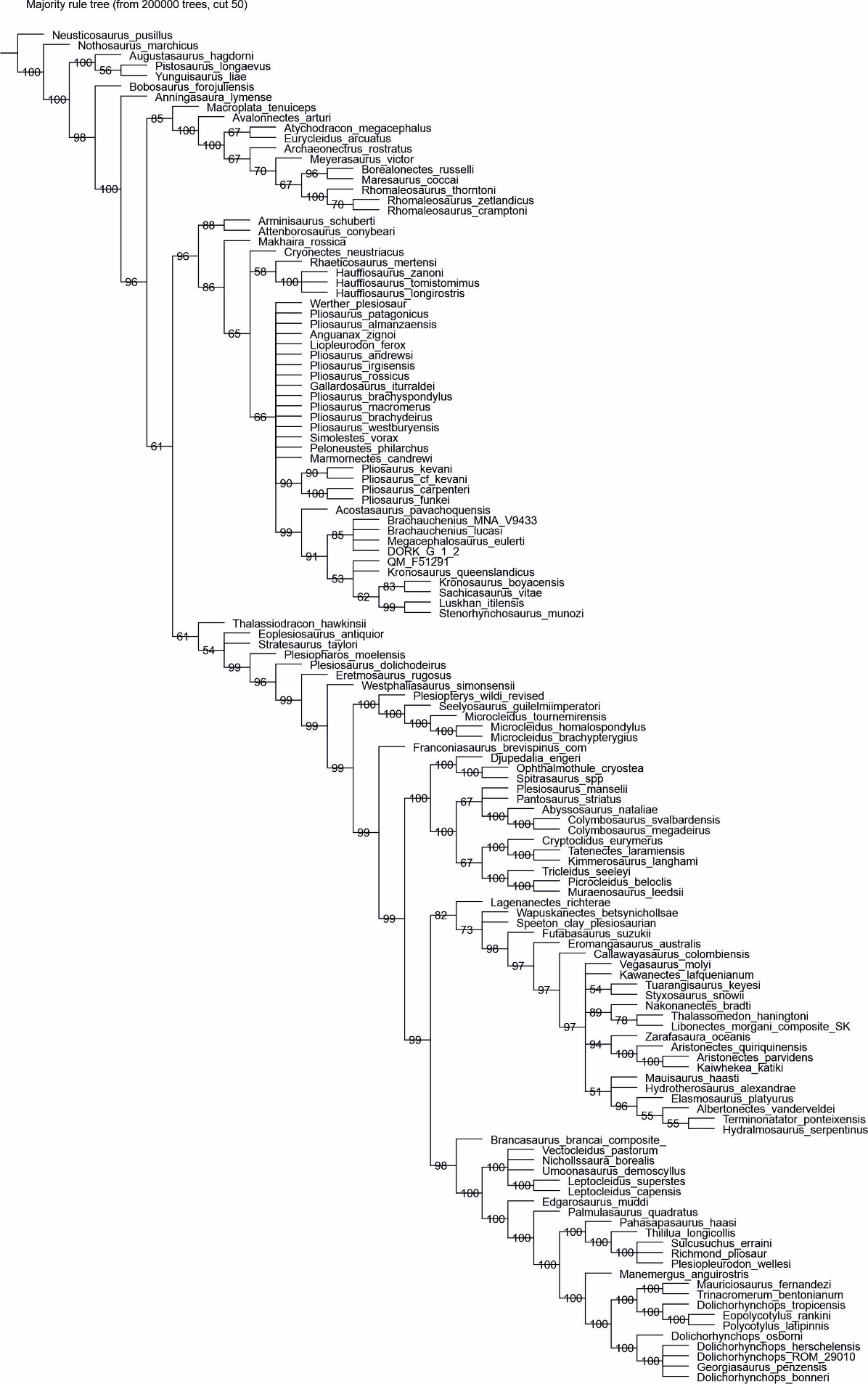


**Figure S3.** Parsimony analysis with implied weighting (*K* = 6). Strict consensus tree.


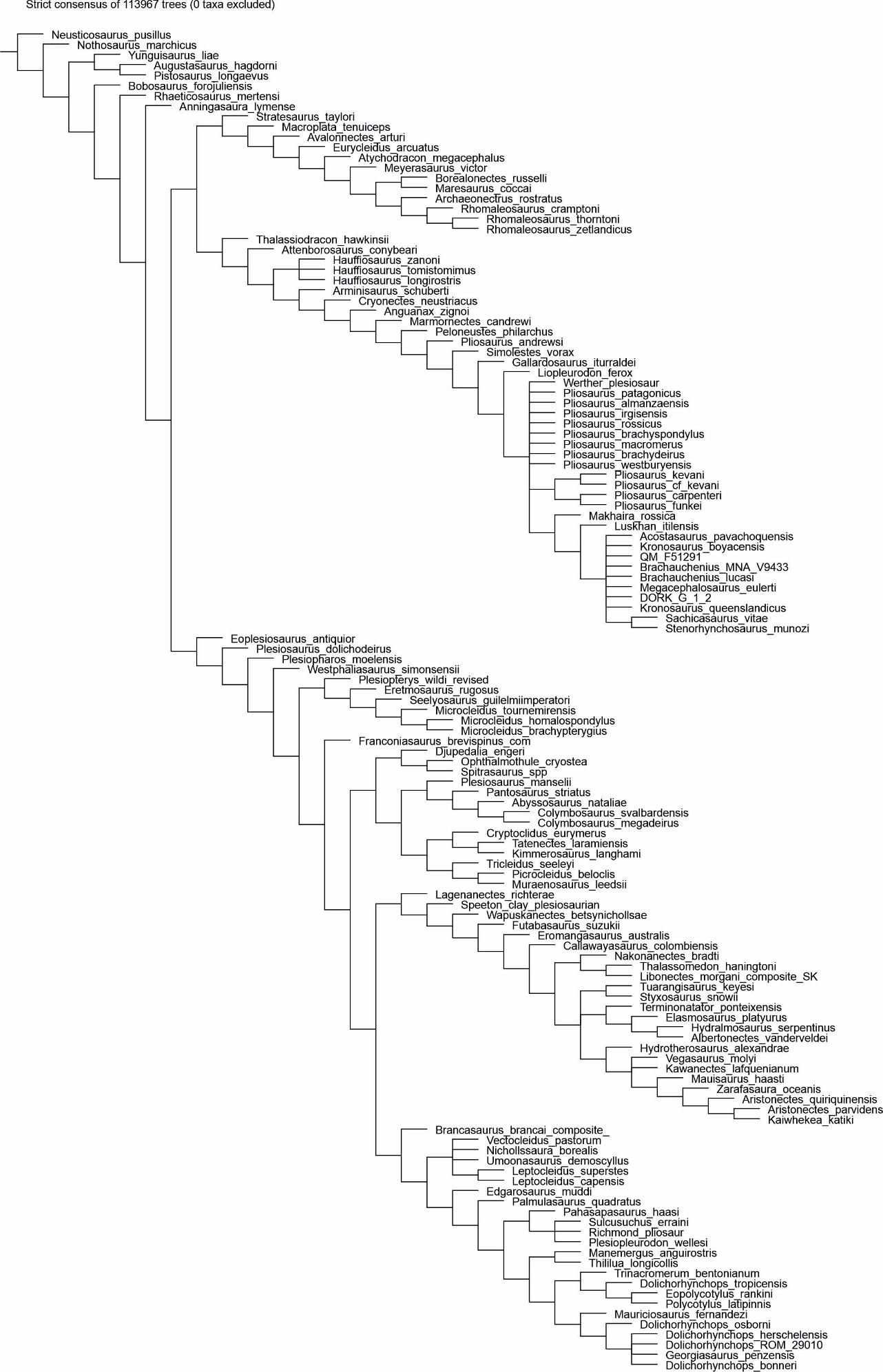


**Figure S4.** Parsimony analysis with implied weighting (*K* = 6). Symmetric Resampling.


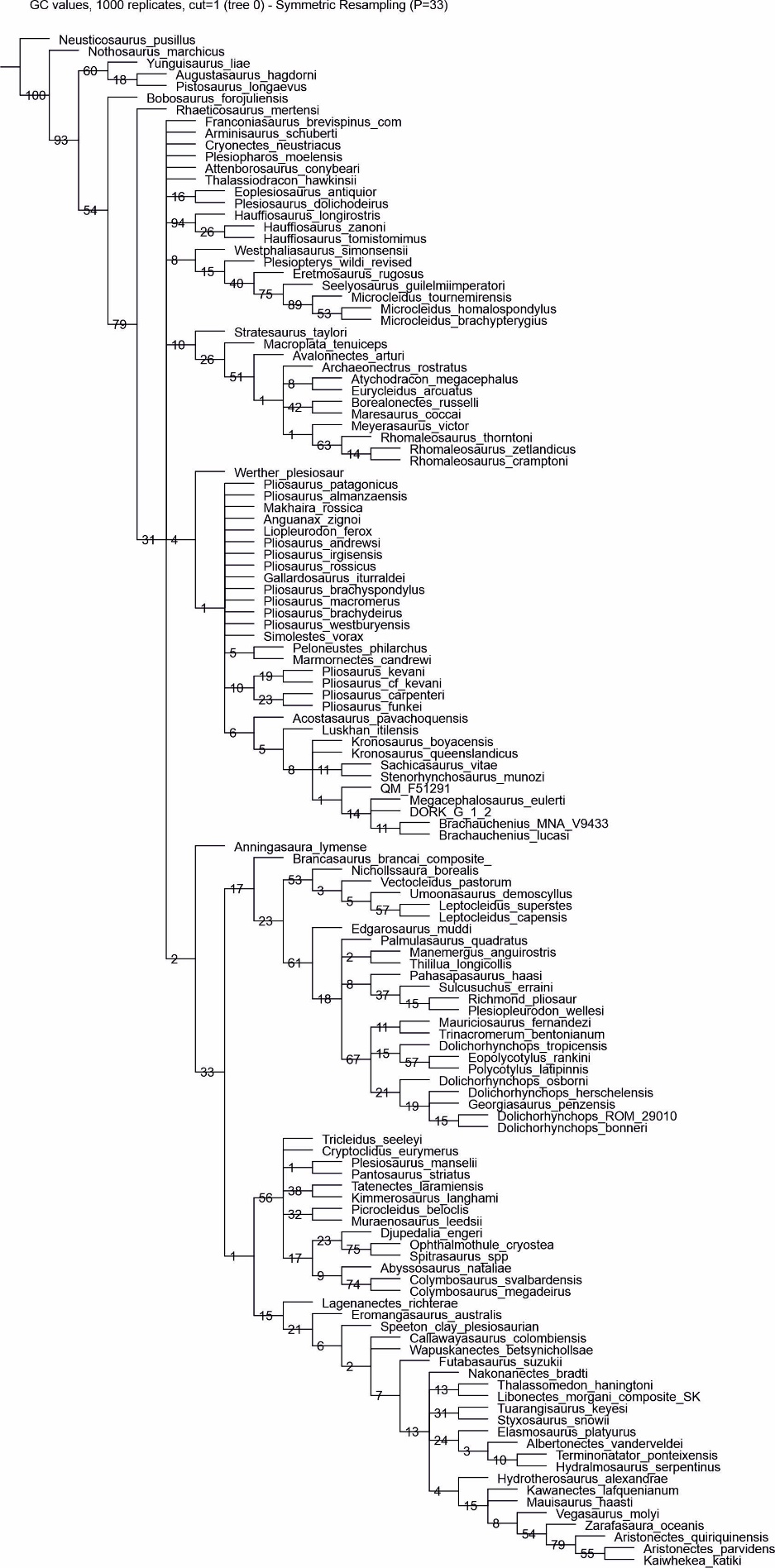


**Figure S5.** Parsimony analysis with implied weighting (*K* = 9). Strict consensus tree.


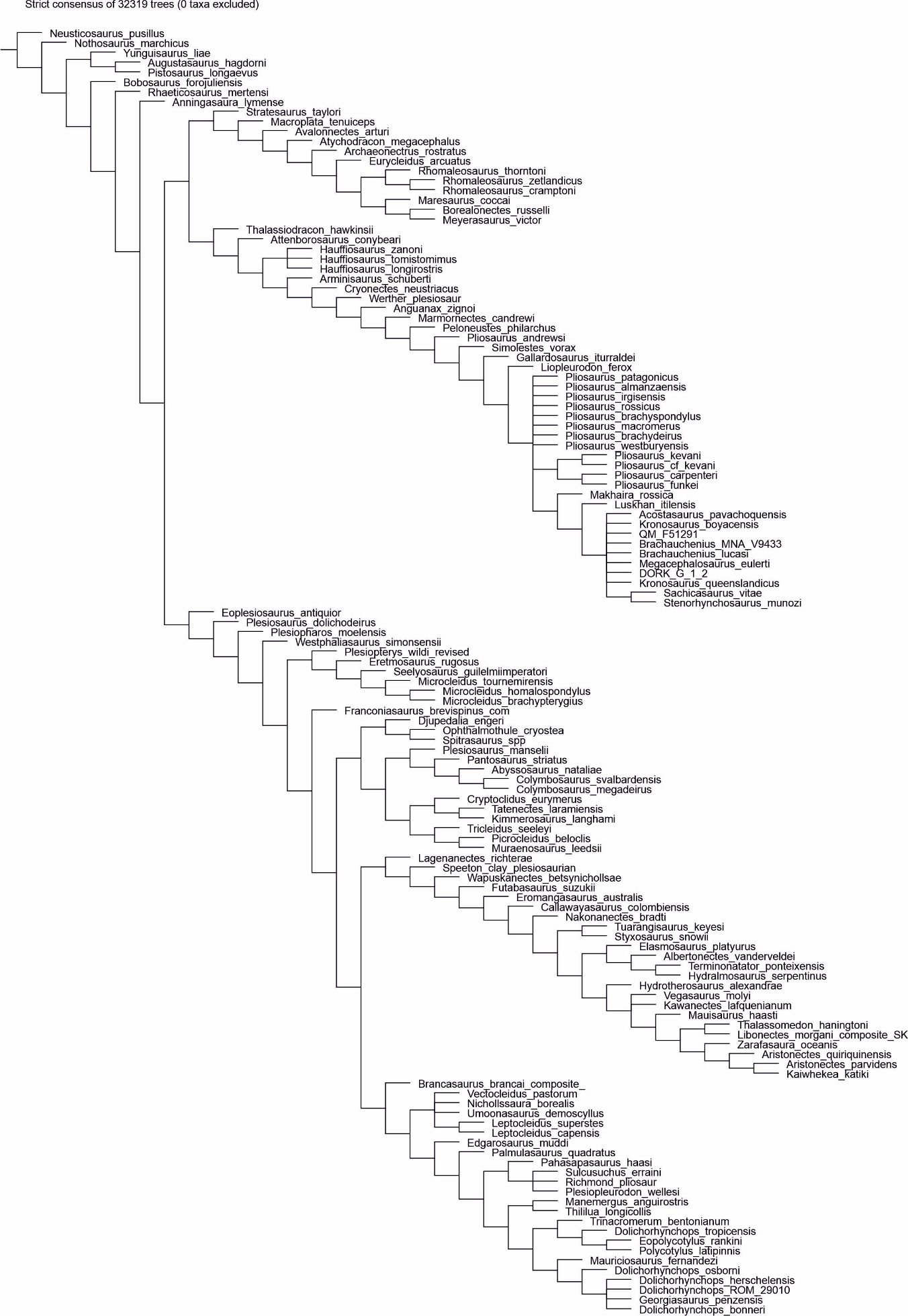


**Figure S6.** Parsimony analysis with implied weighting (*K* = 9). Symmetric Resampling.


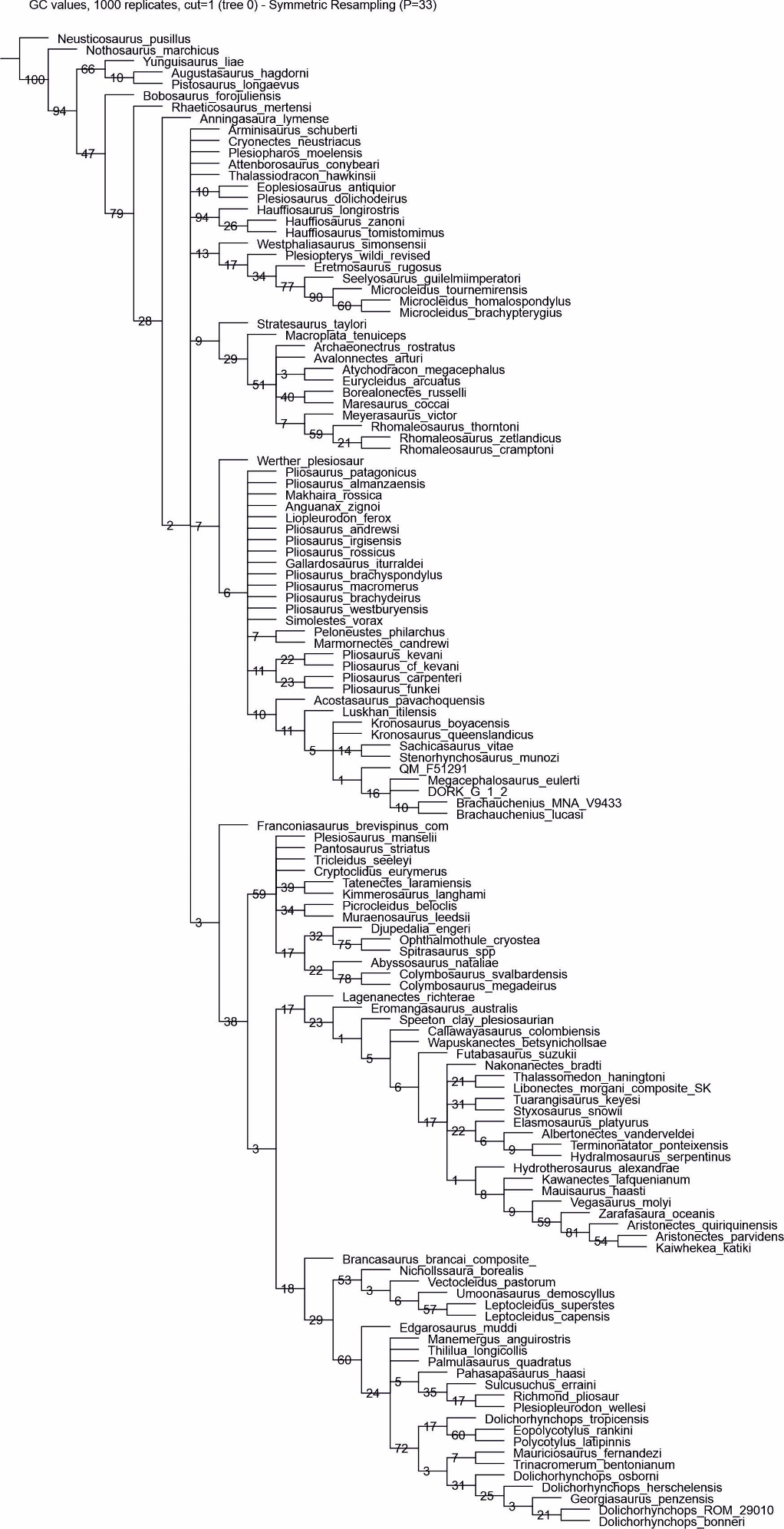


**Figure S7.** Parsimony analysis with implied weighting (*K* = 12). Strict consensus tree.


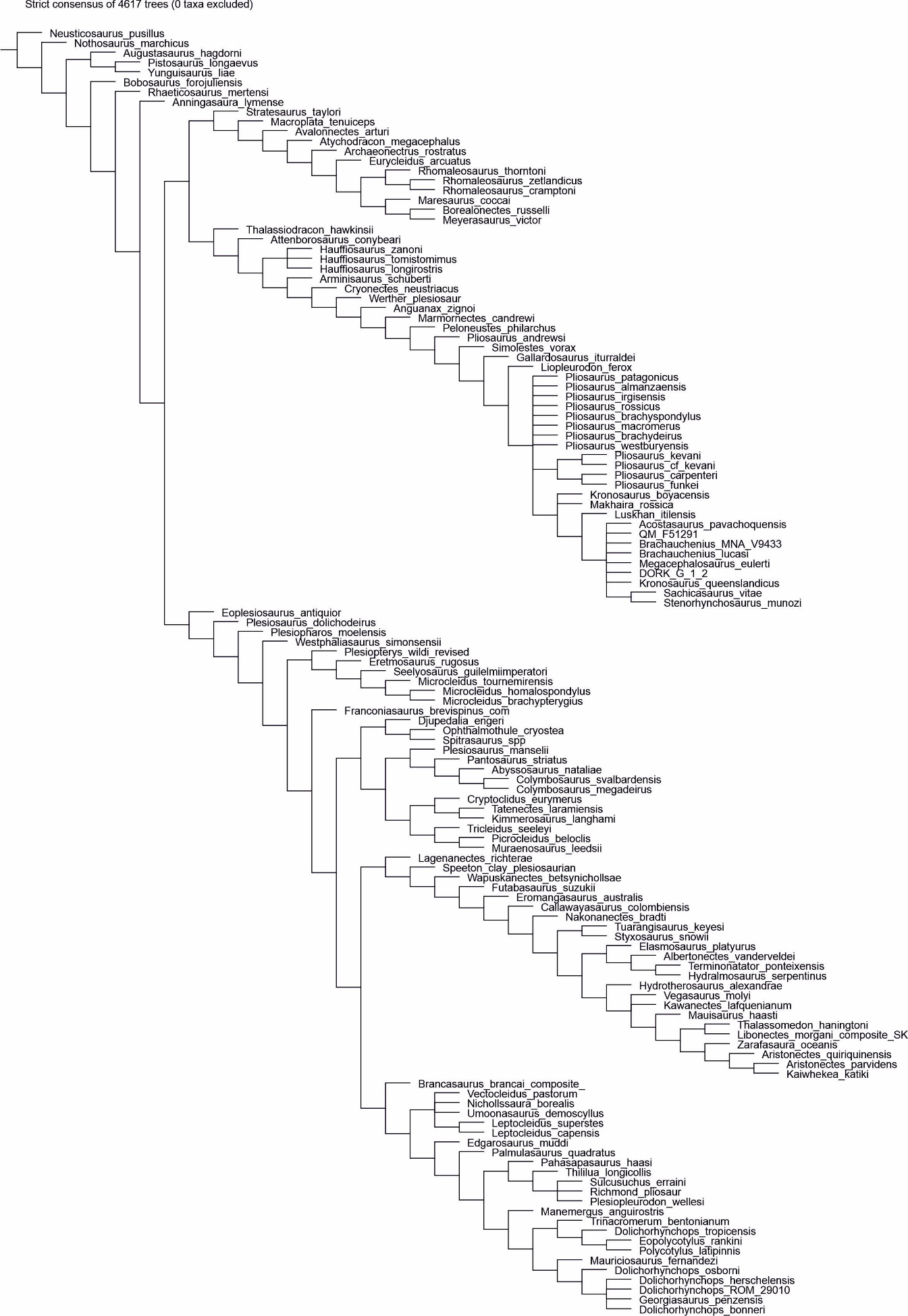


**Figure S8.** Parsimony analysis with implied weighting (*K* = 12). Symmetric Resampling.


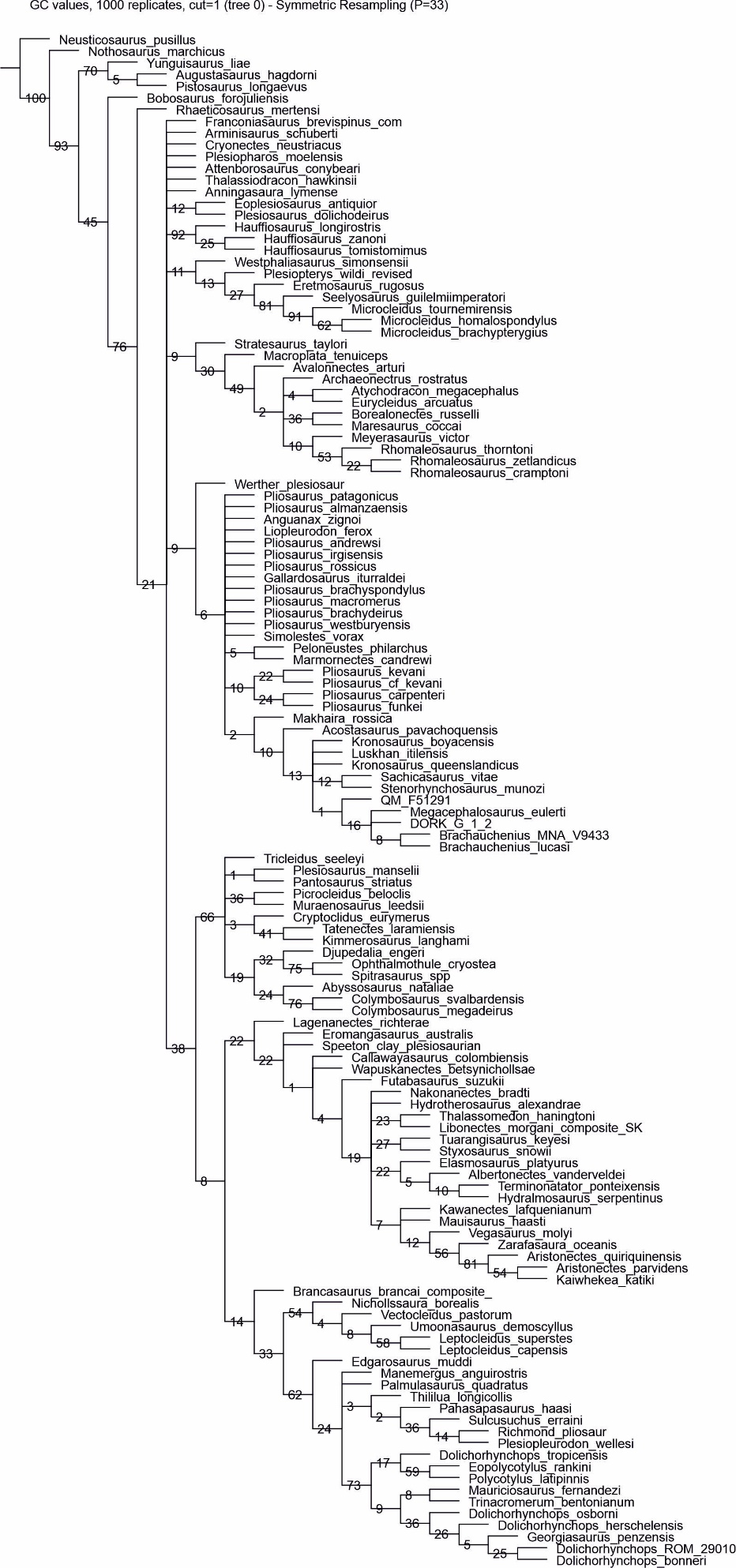

Supplement: Supplemental Information 3 [file peerj-12-18408-s003.docx]
